# The Dichloroacetate Dilemma: Environmental Hazard versus Therapeutic Goldmine—Both or Neither?

**DOI:** 10.1289/ehp.1002554

**Published:** 2010-10-04

**Authors:** Peter W. Stacpoole

**Affiliations:** Department of Medicine, Division of Endocrinology and Metabolism, and Department of Biochemistry and Molecular Biology, College of Medicine, University of Florida, Gainesville, Florida, USA

**Keywords:** cancer, dichloroacetate, glutathione transferase zeta, hereditary tyrosinemia, maleylacetoacetate isomerase, mitochondrial disease, peripheral neuropathy, pyruvate dehydrogenase, toxicogenetics

## Abstract

**Background:**

Dichloroacetate (DCA) is known to environmental scientists as a by-product of water chlorination and as a metabolite of industrial solvents, including Superfund chemicals. In contrast, DCA is studied by clinical investigators for its therapeutic potential in several life-threatening conditions, including genetic mitochondrial diseases, pulmonary arterial hypertension, and cancer. Thus, DCA holds an almost unique position at the interface between environmental science and allopathic medicine.

**Objective:**

I critically reviewed laboratory and clinical pharmacological research on DCA to address questions about the current and future status of DCA in relation to human health.

**Discussion:**

Recent information on the clinical toxicogenetics of DCA is interpreted particularly in light of its use as an investigational drug. Adverse effects from chronic DCA exposure have been identified in several target organs in animals. However, in humans, toxicity has so far been limited to reversible effects on the nervous system and liver. DCA is primarily biotransformed to glyoxylate by the bifunctional enzyme glutathione transferase zeta1 and maleylacetoacetate isomerase (GSTz1/MAAI), which also catalyzes the penultimate step in the phenylalanine and tyrosine catabolic pathway. DCA is a suicide inhibitor of GSTz1/MAAI, which can result in delayed plasma clearance of DCA and the accumulation of potentially toxic tyrosine intermediates. Age and GSTz1/MAAI haplotype can markedly affect the toxicokinetics of DCA in humans and rodents.

**Conclusions:**

I have defined new potential avenues of research that focus on discrete human populations that may be at increased health risk or that may receive increased health benefit from chronic exposure to DCA at both environmentally and clinically relevant concentrations.

To many readers of this journal, dichloroacetate (DCA) has a bad reputation that is mainly the result of its association with Superfund chemicals and other halogenated hydrocarbons of ill repute (Stacpoole et al. 1998b), the findings of numerous animal studies implicating DCA in a variety of moderate to life-threatening toxicities [International Agency for Research on Cancer (IARC) 2004], and the ubiquity of this molecule in our biosphere that makes at least some degree of chronic exposure inevitable (Ammini and Stacpoole 2003; IARC 2004). Despite these concerns, the possibility that DCA might have a positive impact on certain human populations has been evident for half a century (Stacpoole 1989). Over time, DCA’s pharmacological profile has created an interesting dialectic between environmental toxicologists, who consider it to be a hazard to humans, and clinical investigators, who are pursuing its therapeutic potential. Most ironic is the growing controversy over the relevance of DCA to human cancer, in which it is viewed as both a likely cause (IARC 2004) and a possible treatment (Michelakis et al. 2010).

The DCA dilemma has been framed by three broad fields of science: epidemiological studies of populations exposed to environmental levels of measured by-products of water disinfection or Superfund chemicals that are precursors of DCA; mechanism-based toxicological experiments in which inbred rodent strains receive doses of DCA usually vastly higher than those to which most humans are exposed in the environment; and research in which subjects are administered DCA as a drug at doses similar to those used in animal toxicological investigations. Together, the data generated by such research address three questions that are at the heart of the DCA dilemma: Is DCA a significant environmental health hazard? Are certain populations at increased health risk or benefit? What are the important areas of future research that will help confirm, modify, or reject current opinions about DCA’s impact on human health?

Is DCA a significant environmental health hazard? Human populations have been exposed to DCA for generations, primarily by ingesting chlorinated drinking water that may contain up to 160 μg/L (Miller and Uden 1983). Thus, consuming 2 L of municipal water may result in a DCA dose of approximately 2–4 μg/kg body weight in a 70-kg human. However, studies of worldwide exposure to chlorinated drinking water in relation to human disease did not include an assessment of DCA exposure levels (Obolensky et al. 2003). Dermal absorption of DCA may occur when bathing or swimming in chlorinated pools or upon atmospheric exposure, although there are no good estimates of the contribution of dermal absorption to tissue or plasma levels of the compound. DCA is also considered a metabolite of the degreasing agent trichloroethylene (TCE) and related solvents, most of which are released into the atmosphere (Agency for Toxic Substances and Disease Registry 1997). TCE emissions and contamination of surface water vary widely across the United States but correlate broadly with population density and provide an estimated combined median daily intake by the general population of slightly > 13 μg/day (U.S. Geological Survey 2006). Both enzymatic (Lash et al. 2000) and nonenzymatic (Guengerich 2004) catalysis of TCE to DCA have been reported. However, the quantitative significance of either pathway is controversial (Ketcha et al. 1996; Merdink et al. 1998; Templin et al. 1995), which contributes to uncertainty about the relevance of environmental exposure levels of DCA to human health.

Nevertheless, considerable weight has been given to assigning causality to the oxidative metabolites of TCE [see Supplemental Material, Figure 1 (doi:10.1289/ehp.1002554)] in evaluating the toxicity of DCA. Data from population-based pharmacokinetic modeling of the toxicokinetics and risk assessment of TCE and its metabolites (Chiu et al. 2009; Evans et al. 2009) indicate that TCE exposure has been associated with toxicity to the kidney and liver and to the nervous, immune, and reproductive systems. How much of this information is relevant to the assessment of human risk from DCA exposure? Strong evidence from case–control studies indicates an association between TCE exposure and renal cancer in humans (Brüning et al. 2003; Charbotel et al. 2006), but no experimental or clinical evidence is currently available to implicate DCA as a cause of this malignancy. Indeed, no epidemiological investigations have studied the carcinogenicity of DCA in humans. Lee et al. (2003) found a much weaker epidemiological association between environmental exposure to TCE and liver cancer. In this case, however, the relationship between TCE and DCA becomes highly significant, because DCA is unquestionably a liver carcinogen in certain inbred strains of mice and rats (reviewed by IARC 2004; Stacpoole et al. 1998b). Most of the studies that have investigated the carcinogenic potential of DCA have used drinking water or, occasionally, gavage to administer the compound for weeks or months at effective doses usually of 75 mg/kg/day or more [see Supplemental Material, Text A (doi:10.1289/ehp.1002554)]. Regardless of the precise mechanism(s) that underlies DCA’s carcinogenic potential, it is clear that hepatotoxicity in rodents from DCA is obtainable only at doses thousands of times greater than those to which humans are normally exposed from the combined effects of the atmosphere and chlorinated drinking water. This fact, together with current data from population studies, does not support the postulate that exposure to DCA from environmental sources represents a general hazard to humans. Whether chronic exposure to DCA at concentrations encountered in the environment or in the clinic is problematic to select human populations is addressed below.

Are certain populations at increased health risk or benefit? As a medicinal agent, DCA has been administered as the sodium salt in doses ranging from 10 to > 100 mg/kg/day by intravenous and oral routes for a few days to > 10 years [see Supplemental Material, Text B (doi:10.1289/ehp.1002554)]. These clinical studies have reported occasional asymptomatic increases in serum concentrations of alanine aminotransferase and aspartate aminotransferase that seldom exceeded two to three times the pretreatment values of these enzymes and that returned to pretreatment levels within a few weeks after the DCA dose was reduced or stopped (Barshop et al. 2004; Berendzen et al. 2006; Stacpoole et al. 1998b). A more worrisome toxicity of DCA is reversible peripheral neuropathy, an effect replicated in rodents, dogs, and humans (Stacpoole 1989). Clinical features include tingling of the fingers and toes and weakness of the facial and distal muscles of the extremities, accompanied by reduction in nerve conduction velocity in the sural and tibial nerves [see Supplemental Material, Text C (doi:10.1289/ehp.1002554)].

One possible explanation for the hepatic and neurological toxicity of DCA relates to the unusual consequences of its biotransformation. DCA is dehalogenated to glyoxylate by the cytosolic zeta-1 family isoform of glutathione transferase (GSTz1), which is identical to maleylacetoacetate isomerase (MAAI), the penultimate enzyme in the phenylalanine/tyrosine catabolic pathway [see Supplemental Material, Figure 2 (doi:10.1289/ehp.1002554)]. DCA causes a mechanism-based inhibition of GSTz1/MAAI (Anderson et al. 1999), an effect so profound as to significantly delay the plasma clearance of DCA at both environmental (micrograms per kilogram per day) and clinical (micrograms per kilogram per day) exposure levels in humans (Henderson et al. 1997; Jia et al. 2006; Schultz and Shangraw 2006; Stacpoole et al. 1998a) [see Supplemental Material, Text D (doi:10.1289/ehp.1002554)].

Genetic or DCA-induced ablation of GSTz1/MAAI causes accumulation of the MAAI substrates maleylacetone and, presumably, maleylacetoacetate (MAA) (Ammini et al. 2003; Cornett et al. 1999; Lim et al. 2004; Tzeng et al. 2000), although the latter compound is too unstable to be detected by gas chromatography–mass spectrometry. Depending on exposure level, recovery of basal GSTz1/MAAI enzyme activity occurs over days to months after the withdrawal of DCA (Guo et al. 2006; Saghir and Schultz 2002). The toxicological significance of inhibition of MAAI is highlighted by the consequences of loss of function mutations in fumarylacetoacetate (FAA) hydrolase, the terminal enzyme of phenylalanine and tyrosine catabolism. Deficiency of this enzyme causes heredity tyrosinemia type 1 (hepatorenal tyrosinemia), an autosomal recessive disease that has an estimated prevalence of 100,000 persons worldwide (Mitchell et al. 2001). In this disease, accumulation of the substrates for both the hydrolase and MAAI-catalyzed reactions are thought to be responsible for the dramatically increased risk of hepatocellular carcinoma in affected young children, because of the ability of MAA and FAA to form adducts (Lantum et al. 2002). This property is also shared by glyoxylate (Anderson et al. 2004), the product of the GSTz1/MAAI-mediated dehalogenation of DCA.

Another important consequence of tyrosinemia type 1 is the diversion of carbon precursors to succinylacetone, high urinary levels of which are diagnostic for the disease. Succinylacetone is a competitive inhibitor of δ-aminolevulinate dehydratase (enzyme–inhibitor dissociation constant *K_i_* = 0.3 μM; Sassa and Kappas 1983), which catalyzes an early step in heme biosynthesis. Inhibition of the dehydratase causes accumulation of δ-aminolevulinate, a neurotoxin that has been implicated in several peripheral neuropathies, including lead poisoning (see Felitsyn et al. 2008). Increased concentrations of δ-aminolevulinate or subsequent perturbations in heme metabolism or both are thought to be the cause of the neurological manifestations of tyrosinemia type 1, including peripheral neuropathy (Lindberg et al. 1999; Mitchell et al. 2001). Given the effects of DCA on GSTz1/MAAI function, it is noteworthy that increased urinary concentrations of maleylacetone and δ-aminolevulinate have been measured in children who were treated for months or years with DCA. For some of these children, maleylacetone levels approached or exceeded those found in children with hereditary tyrosinemia type 1 (Shroads AL, Langaee T, Coats BS, Kurtz TL, Bullock JR, Weithorn D, Gong Y, Wagner DA, Ostrov DA, Johnson JA, Stacpoole PW, unpublished observations) [see Supplemental Material, Text E (doi:10.1289/ehp.1002554)].

Recently, age and *GSTz1/MAAI* genotype have emerged as important determinants of clinical toxicity from DCA. For example, in a randomized clinical trial of 43 children (mean age at entry, 5.6 years) with congenital forms of lactic acidosis, Stacpoole et al. (2006) found no difference in clinical or biochemical adverse effects, including hematological, hepatic, and peripheral nerve function, between those children who received 25 mg/kg/day of sodium DCA and those who received placebo for 6 months. In contrast, a controlled clinical trial of 30 patients with mitochondrial encephalomyopathy, lactic acedosis, and stroke-like episodes (MELAS) whose mean age at entry was 30 years, ended prematurely because of drug-associated neurotoxicity (Kaufmann et al. 2006). In examining the plasma kinetics of DCA in a representative sample of children and adults from both trials, a striking age-dependent decrease was discovered in plasma DCA clearance that was replicated in rats (Shroads et al. 2008); this finding confirmed and extended earlier observations in rodents (James et al. 1998; Schultz et al. 2002). Furthermore, delayed clearance of DCA in rats was associated with increased urinary excretion of DCA, maleylacetone, and monochloroacetate (a putative neurotoxin and a very minor product of DCA metabolism) and with a decrease in urinary oxalate, an end product of DCA biotransformation [see Supplemental Material, Figure 1 and Text F (doi:10.1289/ehp.1002554)].

Age alone does not account entirely for variability in the kinetics and biotransformation of DCA. The human *GSTz1/MAAI* gene contains four nonsynonymous single-nucleotide polymorphisms, with products that show different activities toward DCA and variable frequencies among ethnic and racial groups (Lim et al. 2004; Stacpoole et al. 2008; Shroads AL, Langaee T, Coats BS, Kurtz TL, Bullock JR, Weithorn D, Gong Y, Wagner DA, Ostrov DA, Johnson JA, Stacpoole PW, unpublished observations). Using 1,2-^13^C-DCA, several researchers were able to clearly segregate “slow” and “fast” metabolizers of DCA, based on GSTz1/MAAI (Shroads AL, Langaee T, Coats BS, Kurtz TL, Bullock JR, Weithorn D, Gong Y, Wagner DA, Ostrov DA, Johnson JA, Stacpoole PW, unpublished observations). Those who metabolized DCA slowly showed markedly delayed plasma clearance and biotransformation, increased excretion of unmetabolized drug [see Supplemental Material, Figure 3 (doi:10.1289/ehp.1002554)], and increased urinary accumulation of maleylacetone and δ-aminolevulinate. Atomic modeling of the effect of *GSTz1*/*MAAI* polymorphism showed that variants associated with slow DCA metabolism induced structural changes in the enzyme homodimer leading to protein instability or abnormal protein–protein interactions. These data suggest that *GSTz1*/*MAAI* haplotype may predict, at least in part, the toxicogenetics of DCA. Moreover, such information may be used prospectively to adjust dosing and mitigate the risk of adverse events from DCA.

Despite the potential toxicity of DCA, its therapeutic potential continues to be investigated. Its principal site of pharmacodynamic action is the mitochondrial pyruvate dehydrogenase (PDH) complex (Stacpoole 1989), a gatekeeper enzyme for regulating carbohydrate fuel metabolism in cells [see Supplemental Material, Figure 4 (doi:10.1289/ehp.1002554)]. Under aerobic conditions, PDH catalyzes the rate-determining step in glucose oxidation by irreversibly decarboxylating pyruvate to acetyl coenzyme A, thereby “priming” the Krebs cycle with glucose carbon. In addition, both the PDH-catalyzed reaction and several enzymes of the Krebs cycle generate reducing equivalents in the forms of nicotine adenine dinucleotide and flavin adenine dinucleotide that are used by the respiratory chain for the reduction of molecular oxygen to water and for ATP synthesis from ADP. PDH is reversibly phosphorylated and inactivated by a family of PDH kinase (PDK) isoforms that exhibit variable tissue expression (Sugden and Holness 2003). DCA exerts differential inhibition on PDKs to maintain PDH in its unphosphorylated, catalytically active form and exerts greatest inhibition (*K_i_* = 0.2 mM) (Bowker-Kinley et al. 1998) toward the ubiquitously expressed PDK2. Because pyruvate is in equilibrium with lactate, it follows that acquired or congenital deficiencies of PDH may lead to mitochondrial energy failure and lactate accumulation. Accordingly, DCA has been used to improve mitochondrial energetics and to mitigate tissue or systemic lactic acidosis (see Stacpoole et al. 1992, 2006 and references therein).

Invoking the same site and mechanism of action described above, several laboratories have reported DCA’s selective proapoptotic and antiproliferative effects when administered *in vitro* to a variety of cultured human tumor cells or to human tumors implanted into immunocompromised rodents (reviewed by Michelakis et al. 2008). The rationale for considering DCA as an anticancer drug is predicted on the well-established finding that solid tumors derive most of their energy from glycolysis, rather than through mitochondrial oxidative phosphorylation, even in the presence of adequate oxygen availability. This phenomenon is called the Warburg effect and is now known to be a consequence of the combined transactivation of glucose membrane transporters, glycolytic enzymes, and PDK (Vander Heiden et al. 2009). Consequently, PDH activity and oxidative phosphorylation are inhibited in most cancers. By inhibiting PDK in tumor cells, DCA stimulates PDH and oxidative phosphorylation, decreases tumor lactate concentration, and initiates a caspase-mediated chain of reactions leading to apoptosis of tumor, but not of host, cells (Bonnet et al. 2007) [see Supplemental Material, Text G (doi:10.1289/ehp.1002554)].

Primary arterial hypertension represents another neoplastic (but nonmalignant) condition in which mitochondrial dysfunction and hyperproliferation of cells lining the pulmonary vasculature lead to increased pulmonary vascular resistance, failure of the right ventricle of the heart, and premature death. In animal models of primary arterial hypertension, DCA has been shown to prevent or reverse mitochondrial damage and cellular proliferation, improve lung function, and increase survival by its ability to stimulate oxidative phosphorylation and induce apoptosis in proliferating cells (Bonnet et al. 2006; McMurtry et al. 2004; Michelakis et al. 2002). Results from clinical trials of DCA in this disease have not yet been reported [see Supplemental Material, Text H (doi:10.1289/ehp.1002554)].

What are important areas of future research? Few chemicals have integrated the disciplines of environmental science and clinical research as effectively as DCA. Recent studies investigating the toxicogenetics of this compound when administered as a drug have important implications for human populations chronically exposed to environmentally relevant concentrations. The findings are particularly germane to persons in contact with water sources contaminated with TCE or other industrial solvents or who are administered drugs that may be biotransformed to DCA (reviewed by Stacpoole et al. 1998b). The recent discovery of a mitochondrial GSTz1/MAAI (Li et al. 2011) indicates that both the biotransformation of DCA and the site of its major pharmacodynamic action, the PDH complex, occur within the matrix of this organelle. This finding raises intriguing questions about whether the stimulation of PDH activity and, subsequently, the respiratory chain by DCA could generate sufficient amounts of reactive oxygen species to cause oxidative damage to mitochondrial *GSTz1/MAAI* and further impair DCA metabolism.

New epidemiological research on DCA should focus more selectively on populations that may be predisposed to DCA-associated health complications because of age or genotype. For example, individuals with an inborn error of the phenylalanine and tyrosine catabolic pathway could suffer additional adverse consequences when exposed to clinically, and perhaps even environmentally, relevant doses of DCA. Individually, these diseases are rare. However, perhaps the most common of these conditions, hyperphenylalaninemia (which includes phenylketonuria), has a worldwide annual incidence that varies widely from approximately 3 to > 300 per million births (Scriver et al. 2001). Another population at potential risk is persons prone to hyperoxaluria. For example, primary hyperoxaluria has a prevalence of > 1 per 10,000 persons and may cause widespread calcium oxalate crystal deposition (Danpure 2001). Such individuals could be at heightened risk for developing calcium oxalate deposits were they to receive clinical doses of DCA over long periods of time.

Likewise, humans who metabolize DCA slowly may also be genetically predisposed to exhibit reduced flux through the phenylalanine and tyrosine catabolic pathway. Chronic consumption of a high-protein diet by such individuals could lead to an accumulation of toxic tyrosine intermediates, with adverse consequences for hepatic, neurological, and other organ system failure. Testing this postulate could involve studies of stable isotope amino acid kinetics in relation to *GSTz1*/*MAAI* haplotype. Finally, careful consideration must be given to age and genotype when conducting future treatment-oriented research with DCA, particularly cancer trials involving adults.

DCA has so far been proven to be neither a major environmental health hazard nor a panacea for human ills. Future clinical and environmental research on this unusual molecule should be focused increasingly on understanding its impact on biological processes in discrete subpopulations of humans with known *GSTz1*/*MAAI* genotype. We continue to move incrementally, but steadily, from puzzled observations about the pharmacology of DCA to established conclusions about its effect on human health.
